# Certification as support for resilience? Behind the curtains of a certification body — a qualitative study

**DOI:** 10.1186/s12913-020-05608-5

**Published:** 2020-08-08

**Authors:** Dag Tomas Sagen Johannesen, Preben Hempel Lindøe, Siri Wiig

**Affiliations:** 1 Department of Media and Social Sciences, The Faculty of Social Sciences, University of Stavanger, 4036 Stavanger, Norway; 2 Department of Health and Nursing Science, University of Agder, 4604 Kristiansand, Norway; 3 SHARE-Center for Resilience in Healthcare, Faculty of Health Sciences, University of Stavanger, 4036 Stavanger, Norway; 4 Department of Safety, Economics and Planning, Faculty of Science and Technology, University of Stavanger, 4036 Stavanger, Norway

**Keywords:** Certification, External assessment, Regulation, ISO, ISO 9001, Resilience

## Abstract

**Background:**

Certification in healthcare often involves independent private sector bodies performing legally required or voluntary external assurance activities. These certification practices are embedded in international standards founded in traditional beliefs about rational and predictable processes for quality and safety improvement. Certification can affect organizational and cultural changes, support collaboration and encourage improvement that may be conducive to resilient performance. This study explores whether ISO 9001 quality management system certification can support resilience in healthcare, by looking at characteristics in the objectives, methods, and practice of certification from a certification body’s perspective.

**Methods:**

One of Norway’s four certification bodies in healthcare was studied, using an explorative embedded single-case design. The study relies on document analysis of the international standards and associated guidances for the performance of certification bodies and thematic analyses of data from 60 h of observations of auditors in three certification processes and nine qualitative interviews with managers and personnel from the certification body. Results from the analyses were compared to identify discrepancies between the written and perceived certification approach and practice.

**Results:**

Standards and guidances for certification embed an elasticity between formal and consistent assessments of nonconformities in organizations and emphasize holistic approaches that brings added value. Auditors were then left with the latitude to navigate their auditing strategy during interaction with the auditees. Members of the certification body perceived and practiced a holistic and flexible auditing approach using opportunities to share knowledge, empower and make guidance for improvement.

**Conclusions:**

ISO certification expects structures and systems to ensure consistent and objective certification processes. At the same time, it embodies a latitude to adopt flexible and context-specific certification approaches, as demonstrated by a certification body in this study, to give added value to the certified organizations. Such an ISO 9001 certification approach may support resilient performance in healthcare by nurturing the potential to respond and learn. These results are important for further development of methods that certification bodies use in the auditing encounter.

## Background

External assessment programs, such as certification, accreditation, peer reviews and inspections are widely used as a regulatory means of assurance, accountability and performance improvement in healthcare [1–5]. Originating in voluntary self-regulation, certification and accreditation programs are increasingly becoming statutory regulatory mechanisms in healthcare [2, 6]. In Norway there are no legally required certification programs in healthcare, but several organizations have voluntarily become ISO (International Organization for Standardization) 9001 Quality Management System certified.

There have been several claims about limited evidence from external assurance mechanisms of the effects upon recognized quality measures in healthcare [7–9]. Two systematic reviews have found no strong effects upon clinical outcomes from certification and accreditation [10] or external inspections [11] in healthcare. Other reviews have shown that accreditation in healthcare might have organizational impact and foster change and instill professional values, stimulate improvement work, and promote organizational and cultural change concerned with quality of care and change in professional practice [4, 12, 13]. Moreover, studies show a positive association with quality and safety structures and hospital outputs such as hospital management and clinical leadership, systems for safety and patient-centeredness [14–16]; and to be effective for organizational change, developing relationships, cooperation and nurturing links between healthcare organizations and other stakeholders [17]. Benefits from accreditation seems to be linked to the motivation for the activities involved in the process [18]. Recent research from Australian hospitals has demonstrated that accreditation supports continuous and systematic quality improvement [19]. However, little is known in healthcare about the approach and methods that external assessment bodies use in their assessment and verification processes, such as auditor’s role repertoire, auditor’s conduct (e.g., inspection or guidance) and assessment practice [4, 20–22].

### Theoretical approach and ISO 9001 certification

We now present our theoretical approach, using resilience to understand ISO certification processes.

The concept of resilience has, in recent years, been applied to healthcare [23–25]. Resilience is defined as “[ …] an expression of how people, alone or together, cope with everyday situations – large or small – by adjusting their performance to the condition. An organization’s performance is resilient if it can function as required under expected and unexpected conditions alike (changes/disturbances/opportunities)” [26]. The resilience literature focuses on the difference between (a) work as prescribed and expected in regulations, guidances, standards, work planning and design, and (b) the work that actually occurs. The former is Work-as-Imagined (WAI) and the latter Work-as-Done (WAD). Supporters of resilience in healthcare emphasize the development of flexible and adaptable local systems, where local knowledge and professionals’ judgments are at the heart of everyday performance [24, 27]. Resilience in healthcare builds upon four potentials — Respond, Monitor, Anticipate, Learn — which need to be managed and supported to bring about resilient performance [26]. The first potential is the ability to *respond* to the situation and know what to do. It is associated with the ability to respond to regular and irregular changes and opportunities, and initiate prepared actions, adjust activities or creating new ways of doing things. The second potential, *monitor,* is knowing what to look for that can improve or diminish organizational performance. *Anticipate* means knowing what to expect and when conditions change. The final potential, *learn,* addresses the factual by knowing what has happened and being able to learn from it. Learning can be specific or institutional [26].

The ISO 9001 standard [28, 29] is a generic norm for quality management systems. It is intended for adaptation to all organizations, from the manufacturing industry to service organizations, such as most healthcare organizations. Organizations can be assessed by an external third-party organization for ISO 9001 certification [30], meaning that it has been recognized for the fulfillment of requirements in the standard. These external assessments are termed *third-party conformity assessment* or *certification audits* and are performed by certification bodies in accordance with the normative standard *ISO/IEC 17021* [31].

**Fig. 1:**
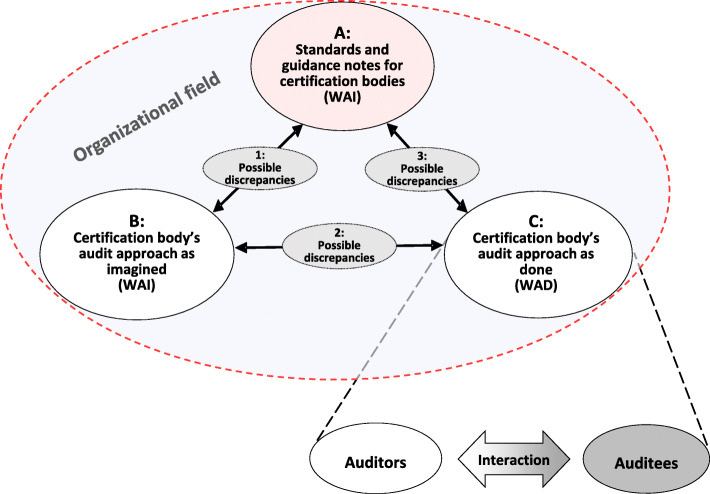
The analytical model

The certification regime involves three essential control components or methods used to affect behavior, and directed at those persons or institutions that seek to be influenced or controlled: *direction* (standard setting) *detection* (information gathering), and *enforcement* (behavior modification) [1, 32, 33]. In ISO 9001 certification, certification bodies use the ISO 9001 standard to *direct* and assess organizations for certification, but the certification bodies themselves have no direct influence on the development of the standard. The certification bodies are themselves *directed* by the international ISO/IEC 17021 standard and related guidances which require certification bodies to manage and keep control of the prescribed and practiced approach to *detection* and *enforcement* in certification auditing processes. The distinction relates to the resilience perspective of Work-as-Imagined and Work-as-Done [27], where WAI includes both the ISO/IEC normative standards for certification processes and the certification bodies’ own prescriptions and perceptions of certification processes. WAD, in contrast, describes the practices that unfold in certification encounters. Figure 1 (explained in the methods section) illustrates this perspective.

The main assessment activities, detection and enforcement in ISO 9001 certification are processed by auditors interacting with the certified organizations in on-site certification audits. A classical distinction used to describe the detection and enforcement styles in regulatory encounters, is the one between compliance or deterrence [1, 5, 33–35]. Compliance is prospective, and focused on preventing harm, forming closer relationships, cooperation, support, education, improvement work, and the use of formal sanctions only as the last resort. Deterrence is more retrospective, focused on detecting violation, distant relationships, formal processes, noncompliance, and extensive use of formal sanctions. There is no general agreement on what approach or style to external assessment in certification serves the certified organizations the best [1].

Different certification approaches may support resilience in healthcare organizations in different ways. No studies to our knowledge have previously explored the relation between certification and resilience and how certification processes can support resilience in healthcare.

### Aim and research question

External assessment in healthcare affects organizational and cultural changes, support, collaboration and encourages improvement. Such effects may be fruitful in terms of resilience. This study explores the characteristics of approaches to ISO certification and discusses whether these approaches can support resilience in healthcare. The following research questions guided the study.

1. What auditing approach for certification bodies is embedded in standards and guidance notes for ISO 9001 certification?
2. How do managers and auditors of a certification body perceive and practice the certifications?

The study reports on approaches to certification processes and practices expressed in international ISO certification standards and as seen by auditors and managers in a Norwegian certification body. The paper contributes to our knowledge of the characteristics and flexibility in external reviews that might be important in further development of the ISO certification and external assurance mechanisms.

## Methods

### Design

This study uses an explorative embedded single-case design using several sources of evidence [36, 37]. The case study is based on certification processes implemented by one of the four certification bodies accredited to perform ISO 9001 certification of healthcare organizations in Norway.

### Sources, recruitment, and data collection

The study relies on data collected from document analysis of international standards and guidance notes related to ISO 9001 certification, and qualitative semistructured interviews and observations related to the certification body’s approach to certification (Table 1). The certification body identified managers and certification teams as bases for data collection by interviews and observations. Hospitals collaborating with the certification body were contacted by the author DTSJ. The standards were obtained through ISO’s electronic distribution platform, and the guidance notes were openly available and downloaded from the ISO’s web page.

**Table 1:** Data Sources and Collection

System level	Data sources	Data collection	Location	Duration
International/national (Norway)	6 ISO/IEC standards 55 AAPG and APG guidelines	Document analysis		
Certification body Organizational/individual	3 lead auditors in 3 separate conformity assessments	Nonparticipant observation	1 Hospital 1 Clinic for internal service 1 Emergency department	3 days 2 days 3 days (About 60 h)
Certification body Organizational	5 lead auditors 4 leaders/administrative personnel	Semistructured interviews	Certification body, central office	45–75 min per interview

Data collection took place in three stages. In the first stage, we collected data from the international normative standards and guidance notes for bodies providing ISO 9001 certification. The standards include the ISO/IEC 17021:2011 standard for certification bodies performing ISO 9001 certification and its related standards relevant for this study (Table 2). We also collected data from 55 guidance notes from two international auditing practice groups, constituted as informal groups of experts and practitioners active in the development of the official auditing standards. These were the ISO 9001 Auditing Practices Group (APG)^1^ and the Accreditation Auditing Practices Group (AAPG).^2^

**Table 2:** Standards and Normative References Included in this Study

ISO/IEC 17021:2011	Conformity assessment: Requirements for bodies providing audit and certification of management systems
ISO/IEC 17000:2004	Conformity assessment: Vocabulary and general principles
ISO 19011: 2011	Guidelines for auditing management systems
ISO/IEC Guide 60	Conformity assessment: Code of good practice

In the second stage, we explored certification practice by conducting nonparticipant observations of three lead auditors in three third-party conformity assessments (certification audits) in two hospitals. The first observation was in a clinic for internal service. The second was in a hospital where the objective was to certify the total management system and delivery of specialized health services of a hospital. The third observation was conducted in an emergency department.

The first author followed the lead auditors during on-site audits for 60 h. The observations followed an observation guide focusing on the conduct of the lead auditors during their interviews and conversations (assessment process) with members of the certified organization. Topics covered were interaction and communication, methods of interview and personal style. All observation notes were taken openly and guided by an auditor typology framework [38]. The auditor styles observed were presented in a separate article [39].

In the third stage, nine semistructured interviews (Table 1) were conducted with the lead auditors (five people), and managers and administrative personnel (four people, who were also lead auditors) from the certification body. The interviews lasted 45–75 min and were conducted at the informants’ workplace. The interviews centered upon three themes in the interview guides (see Additional files 1 and 2): 1) the informants’ role, the organization and their approach to ISO 9001 certification; 2) the certification process and regime; and 3) ISO 9001 certification and regulation in healthcare. Open questions were used to make the informants recall and tell detailed stories about the topics addressed. Questions were often followed by either scripted or ad hoc probing questions. All interviews were audiotaped and transcribed verbatim.

### Analytical framework and data analysis

Our analytical framework represents central elements in the certification process and the involved organizations in the healthcare setting (certification body and the certified hospitals). The purpose of the framework is to guide the analytical process by assessing the relationship between the auditing approach embedded in formal standards and guidances (research question 1) and how managers and auditors perceive and experience and practice the certification (research question 2). Figure 1 presents the framing.

In Fig. 1, the objectives and methods for certification as defined in standards (normative references) and guidance notes (A) gives scope of opportunities for certification practices and are perceived and translated by certification bodies (B). Certification bodies also perform the certification activities (C) where auditors access and interact with the certified healthcare organization in the auditing encounter. Ideally, elements A, B and C are harmonized. Numbers 1,2 and 3 in Fig. 1 represent possible discrepancies among the elements. The model assumes that the certified organization also assesses, from an internal perspective, its own documented quality management system and its consistency with the ISO 9001 standard. The two assessments are then matched, discussed and negotiated in the interaction between auditors and the auditee to identify nonconformities or areas in need of improvement.

To explore characteristics in approaches to ISO certification, and be able to compare elements A, B and C in the analytical model the data related to each element was subject to theoretical (deductive) thematic analysis [40, 41]. Two broad a priori themes were applied underpinned by the theoretical opposites of deterrence and compliance approaches to regulatory enforcement [1, 34]. This was operationalized as: (a) *assessing conformity against requirements*, focusing on retrospective auditing practices (detect noncompliance, control, provide formal processes and distant relationships) and (b) *quality improvement work*, focusing on prospective auditing practices (offer guidance, educate, transfer experiences and give advice). The two a priori themes were applied to the thematic analyses described in the next two sections.

To analyze the data according to research question 1, the auditing approach embedded in certification standards and guidance notes, a document analysis was performed by an iterative process combining content analysis and thematic analysis [42]. The analytical process included a superficial examination (skimming and summative content analysis), followed by thorough examination (reading and rereading, reading concepts in context and thematize) and finally interpretation. The qualitative research software NVivo 10 were used for a summative content analysis [43] of the guidance notes. This analysis first included a word frequency query of all words with a minimum of four letters grouped with stemmed words; this led to 1800 words being identified. Second, the results were reviewed and words that were prospective and related to development work were used for a text query within all the guidance notes. These words were *guide*, *utilize, encourage, stimulate, instruct, recommend, suggest, propose, warn, consult, assist, advice, support, give*, and *help*. Finally, the query was spread to a broad context within the guidances, and the contents of the text were then subjected to thematic analysis combined with the content of the ISO/IEC 17021:2011 standard and its related standards. The document analyses identified “work as imagined” in standards and guidances for certification.

To analyze the data according to research question 2, how managers and auditors experience and practice certification, all the data material from the observational field notes and interviews was subjected to thematic analyses [40–42] using the a priori themes. NVivo 10 was used to explore and thematize the interview data. A reflexive approach was used for the analyses, drawing attention to the narratives (stories) [41] that contoured an auditing orientation towards either strict retrospective assessments of conformity to requirements or prospective quality improvement approaches. These analyses identified “work as imagined” by managers and auditors and “work-as-done” in certification practices.

Finally, to compare findings that addressed research question 1 and research question 2, the results from all analyses were reflexively compared to spot discrepancies [37] among A, B and C in the analytical model (Fig. 1). To ensure trustworthiness, we conducted a member check with the certification body.

## Results

In this section we first present the results related to research question 1, the certification auditing approach embedded in standards and guidance notes for certification bodies (WAI) which relates to element A in the analytical model (Fig. 1). We then apply the results to research question 2. First, we explain how managers and auditors of the certification body perceive certification processes (WAI), which relates to element B in the analytical model. We then turn to the practice of certification audits (WAD), associated with element C in the analytical model.

### Element a: approach to certification in standards and guidance notes for certification bodies (WAI)

The standard ISO/IEC 17000 outlining the vocabulary and general principle for conformity assessments in general, defined three functions for conformity assessments: *selection, determination,* and *review and attestation*. *Selection* consists of planning, preparation and specifying the requirements and audit criteria for certification. *Determination* is the development and collection of information regarding fulfilment of specific requirements, such as audit activities. *Review and attestation* are the final stage of checking evidence of conformity before deciding on certification. These functions were recognizable in the ISO/IEC 17021 standard, regulating specific certification processes, which defined the overall objective for certification as “[…] to give confidence to all parties that a management system fulfils specified requirements.”

The ISO/IEC 17021 emphasized that certification bodies and auditors should build confidence and trust through a practice rooted in impartiality, consistency, competent assessments, and decisions based on objective evidence. For certification bodies to do this, the ISO/IEC 17021 detailed requirements for the certification bodies’ organizational structures and their management of impartiality, resources, competence, information and the specific certification processes. Related to formal stages of certification auditing programs and processes, the ISO/IEC 17021 referenced the generic ISO 19011 standard for auditing management systems. Both standards emphasized a consistent audit approach that focused on retrospectively assessing and detecting noncompliance with the requirements of a management system. For example, the standards include almost identical formal clauses with requirements for the stages in on-site (the location of the certified organization) audits such as *conducting the opening meeting, communication during the audit,* [assigning roles and responsibilities of] *observers and guides, collecting and verifying information, identifying and recording audit findings, preparing audit conclusions and conducting the closing meeting*.

Processes that involve human interaction between auditors and auditees in on-site audits, such as interviews- and observation to collect and verify information, were described in short terms or became implicit in the formal stages of the audit processes, such as when confirming, reporting, explaining, introducing and presenting. Interactive activities between auditors and auditees were briefly and explicitly mentioned to *discuss* and *resolve* audit finding and conclusions. An annex in the ISO 19011 gave some informative guidance about conducting formal individual interviews during on-site audits.

In general, the ISO/IEC 17021 and the ISO 19011 standard had a retrospective approach where assessment of the certified organization’s management system activities, processes and products or services, and the extent of conformity to certification requirements were the main subject. Descriptions of prospective auditing approaches, such as support, transfer of experiences or giving advice were almost absent in the standards. According to the ISO/IEC 17021, “The audit team may identify opportunities for improvement but shall not recommend specific solutions.” The standard reiterated the threats to impartiality for certification bodies involved in certification processes. Such threats may come from certification bodies doing management system consultancy work to the certified organization, such as giving advice, describing causes of nonconformities or being involved in improvement work, or when certification bodies are being too familiar with or trusting of auditees instead of seeking audit evidence.

Comparing the guidance notes for certification with the certification standards, we found a more prospective orientation in the notes as described in the next section.

### Guidance notes on ISO 9001 certification

Guidance notes from the Auditing Practice Group (APG) and Accreditation Auditing Practice Group (AAPG) communicated, in principle, that all audits should add value or be useful to the auditee. To do this, APG suggested “[…] a ‘holistic’ approach to evidence gathering throughout the audit, instead of focusing on individual clauses of ISO 9001” (ISO & IAF, 2009, How to add value during the audit process).

It was emphasized that the requirements in the ISO 9001 standard must not be considered a “tick-off” scheme, either by the auditor or the auditee. The auditors should look not only at compliance but also at the effectiveness and benefits of the implemented management system.

Instead of simply looking for formal compliance with the requirements of the standard, auditors should look at the real effectiveness of the management system and identify the benefits that the adoption of the system give to the organization and to its clients. (ISO & IAF, 2008, Added value certification audit versus consultancy)

According to the guidance notes, auditors should furthermore be process- and result oriented, instead of stressing procedures and records, to maximize the possible added value. Adding value was related to making the ISO 9001-based quality management system more useful for the organization. As a further means of adding value, certification audits should provide “[…] information to top management regarding the organization’s ability to meet strategic objectives; by identifying problems which, if resolved, will enhance performance; [and] by identifying improvement opportunities and possible areas of risk” (ISO & IAF, 2009, How to add value during the audit process). This meant that auditors should acquire some understanding and be sensitive to the maturity of the quality management system and the quality culture in the certified organization, in order to modify the auditing approach and reporting of audit findings.

The guidance notes did not include much advice on performing formal interviews or observations, although it did encourage interactive processes in auditing practices.

[…] open discussions with people who are primarily responsible for management of the organization could allow the effective use of audit resources and time and may provide major benefits for the organization. (ISO & IAF, 2008, Added value certification audit versus consultancy)

The guidance notes focused upon prospective approaches in which the improvement of the management system was central to audit practices. Auditors were not to act as consultants by giving advice or explaining “how to solve a nonconformity” situation. However, stimulating improvement should be encouraged:

[A] correct approach to the handling of nonconformances is for auditors to encourage the auditees to find their own solution, by raising questions and stimulating understanding and awareness, but not providing direct advice as to how problems should be solved. (ISO & IAF, 2008, Added value certification audit versus consultancy)

According to the guidance notes, the audit reports should, if possible, go beyond descriptions of mere compliance of audit requirements and identify opportunities for performance improvement but without offering specific solutions.

### Element B: certification body’s audit approach as imagined

The certification audit approach perceived by managers and auditors in the certification body was largely oriented towards a prospective auditing approach.

The informants from the certification body focused on certification as an ongoing process. The certificate in itself should not be the main objective, but the process should bring added value and inspire internal improvement processes in healthcare organizations. The informants used terms like “driver for change,” “added value,” “improvement,” “review the system” and “to have satisfied customers (or patients)” when describing the objectives of the certification processes. They also reported that the certified organizations seldom focused on the certificate but were more concerned with improving their system.

What I'm saying is that the certificate itself is not so important to us, but it's more for you. It's proof that someone has conducted a review and shared their experiences of the journey with you. And now you have the foundation in place. (− 67)

Most informants considered the ISO 9001 standard suitable for the healthcare context, while others described it as one of many suitable standards. There was a consensus that the auditors should focus on the healthcare organizations’ own processes, and less on prescriptions and detailed requirements. An experience among the informants was that the quality management systems in healthcare were often novel, lacking familiarity with management concepts mentioned in the ISO 9001. As a consequence, audit activities had to prioritize achieving “minimum certification standards” and identify significant risks at the expense of facilitating continuous improvement processes. Another concern was that the auditors often needed to do guidance on integrating daily practices into the quality management system.

I constantly try to find out what they are good at and how they can improve, by using what they have in a better way. Simply, cleaning up their own house. It's too much of everything, and maybe they lack what's important — missing the overall perspective many times. (−72)

A general view among the auditors was that the certification process was capable of nurturing local processes in healthcare organizations by enhancing awareness for improvement of processes. One effect often mentioned was the certification processes’ contribution to reducing the number of procedures, or to making the procedures more functional. Some of the informants complained that there was a misconception that the ISO standard was overly complicated and required many procedures. As one informant explained:

It is simply process thinking. But soon you [the hospitals] spend a lot of time looking at paper and bureaucracy, and take the focus away from patients, in order to build a [quality] system. It is a great challenge for us that it [the standard] is known in many countries as: “ISO 9000 - It's just paperwork.” (−76)

Descriptions of the auditing process as purely a collecting of evidence of conformity to requirements in ISO 9001, or performance as in standardized observations and interviews, were almost absent in our interview results. The informants focused on improvement work with the purpose of bringing the organization to an acceptable level of compliance with internal and external requirements, and to becoming certified. Focusing on the minimum level of conformity during certification practice did not match the preferences of the informants. It was clear that they preferred (and considered it their job) to persuade organizations to improve their management system beyond the minimum level of requirements. The informants also emphasized the importance of continuous follow-up from audits, the yearly surveillance audits, and re-certifications every third year to nurture improvement.

The auditors described their position along a continuum from a proactive role, facilitating organizational development towards a reactive role of “compliance with rules.” They were clear about not giving advice, because they might come back later and audit their own solutions. In this context, advice means suggesting specific solutions to problems. Giving examples from other organizations, transfer experiences, or offering solutions that might be fruitful for the organization to discuss or choose from, was not considered “advice.” The informants often experienced such information as what the organizations mostly valued, and they often discussed different solutions. Even good experiences from other sectors were considered valuable for transferring into healthcare organizations.

The certification body had an internal management system consisting of the procedures, checklists and templates for the management of certifications, which intended to meet the requirements in the conformity assessment standard ISO/IEC 17021 for certification bodies. This system was considered important for the reliability and consistency of the certification process. The informants emphasized three key elements to ensure reliability in their approach to certification: the certification body’s internal written routines, templates and checklists for the different stages of certification; the internal control routines that were independent of the respective auditor; and the internal program for competence and regular calibration of auditors. To gain consistency, the certification body’s internal routines and prescriptions defined structures for the certification processes before and after the on-site audits, but they were less concerned with prescriptions for the on-site audit activities.

Many people think that it’s the standard we are auditing according to, but if we take an audit, it's a very small... We do not walk around with the standard [...] So in that way the standard’s requirements are used much less than you might think. (−73)

When considering reliability issues in the auditor-auditee encounter, the informants highlighted the importance of the competence and experience that the individual auditor brought with them and stressed the importance of having auditors with knowledge of the healthcare field in the audit team. Differences in performance among auditors were acknowledged and not seen as a threat to the certification practice. It could even be worthwhile for the auditee to change auditors for several reasons: because a relation over a longer period may threaten the independence of the certification body; because human relations may not always fit; or because one auditor sometimes see opportunities or nonconformities in organizations that another does not. It was pointed out that an audit is only a snapshot of an organization, based on sample controls on parts of the management system.

It has happened - but not very often - that an auditor has been to a place and not seen anything, and then the next auditor comes in and sees a lot of noncompliance and many shortcomings. (−74)

There were differences in the way that auditors approached audit findings and communicated them to the auditee. Defining “observations” or nonconformities was expressed as an important formal part of the certification practice. An observation could lead to a nonconformity if allowed to continue, or a condition without enough evidence to confirm that it constituted a nonconformity. At the same time, most of the informants seemed willing to adapt to organizational circumstances and negotiation when they considered different forms of responses to audit findings. Improvement seemed to be a bearing principle to such considerations. Responses could be to reduce the number of nonconformities by merging “small” nonconformities into larger ones. As one informant described:

Counting nonconformities is never a good indicator. I merged 20 and made two [...] Two big ones can be much more serious than ten small ones. (−70)

Another strategy could be to strengthen the response by giving an observation as a “wake-up call” to motivate the auditees to stretch themselves. According to one informant:

What we often do is try to increase the level a little all the time. Perhaps giving them an observation. Shake things up by giving them an observation and saying: “Assess, then you'll get even better.” (−73)

### Element C: certification body’s audit approach as practiced

Observation of the certification practices identified that the auditors were largely oriented towards a prospective auditing approach. The assessments of conformity to the ISO 9001 standards were interwoven in dialogues and interactions with the auditees.

During the opening meeting in all three on-site certification audits observed, the lead auditors systematically explained how the assessment activities would be carried out. During the meetings it was also emphasized that the audit process was not only an assessment of conformity to the ISO 9001 standard, but also a process intended to encourage improvement of their quality management systems. They all mentioned the importance of the certification process bringing an added value to the hospitals.

We found some differences in the way the lead auditors used pre-planned templates to guide the audit process during dialogues and interviews with the auditee. Two of the lead auditors used a template to highlight topics from the ISO 9001 standard, and to keep track of the conversations with the auditee. The template was also used for systematic or sporadic recordings of the audit findings. The third lead auditor who was observed did not use a template during interviews and dialogues with the auditees. None of the lead auditors used the ISO 9001 standard as a tick-off scheme or referred to specific requirements in the standard. The standard was hardly mentioned during the audits observed, and most often only if the auditees asked for references to relevant requirements in the ISO 9001 for the issues at hand. The lead auditors did not use structured pre-planned questions in their interviews with the auditees, but rather opened for dialogue and discussions. They often used open-ended questions to stimulate self-reflection on the issues at hand. There was always more than one person from the certified organization represented during the planned interviews and dialogues with the auditors. Only some short ad hoc individual conversations between auditors and auditees were observed in some of the on-site walkarounds in the certified organizations.

Our results showed that nurturing local processes was important for promoting improvement and efficiency and for raising awareness. Only a few of the many challenging issues about the hospitals’ quality management system, raised in the conversations between auditors and auditees, were considered as nonconformities. The following three examples show how local awareness of improvement possibilities becomes inherent in conversations between auditors and the auditees about issues related to the hospitals’ quality management system. None of the issues in the examples were reviewed as nonconformities to the ISO 9001.

In the first example, the auditor asked how the handbook communicated laws and regulations, and followed up with a rhetorical question: “How and where would a nurse in a department find or try to find these [laws and regulations]? – [you should] think from the bottom up.” Then, two departments presented examples from their procedures. The first department received positive feedback from the auditor because it had operationalized only the parts of regulations that pertained to the procedure. The second department had provided a long list of references to many laws and regulations in their procedure, but not operationalized it into practical useful information to the employees using the handbook. The auditor responded by repeating, “Think from the bottom up. What is relevant for the employees?”

In the second example, an auditor praised an orthopedic department’s internal annual report as one of the best the auditor had ever seen. The manager (who was also the chief physician) had on his own initiative, for years, systematically registered data from their activities, analyzed it and generated annual internal reports. The report was shown during the audit almost by coincidence, because the manager did not see it as part of the organization’s quality management system, and therefore thought it was not valid for the quality management certification audit. The department’s annual reports were considered more useful for departmental activities, than most of the data collected for the hospital official reports. The auditor responded by emphasizing the importance of the annual internal report, both because it was expected by the ISO 9001 for the department to give inputs to the hospital management, but mostly for organizational control and learning and as a model for other departments. The auditor made certain he would express to the hospital’s top management their responsibility to ensure that they received the best accessible inputs to their management review. The top management review is a requirement in the ISO 9001. The manager was delighted but was not sure if the internal report would be considered legitimate by the hospital management, since it was not part of the mandatory performance report.

In the third example, the auditor was performing a recertification audit of a hospital’s emergency department. Part of the agenda was to review if the written agreements between the emergency department and the internal support departments had been followed up by both parties since the last surveillance audit (the yearly audits between the three years full certification and recertification cycle). When visiting the technical department, the auditor asked questions related to the department’s use and implementation of the documented risk assessment. The manager admitted that risk assessments were often carried out and used to receive funding for improvement. “What about revision of the risk assessment when actions have been taken?” the auditor asked. The manager answered that the risk assessments were seldom revised when different risk related actions had been taken. By the end of the auditor’s visit, the department manager concluded, “I have learnt one thing today: we should make the risk assessments more ‘alive.’”

When the same auditor visited the department for purchasing and supply, one of the topics discussed was how the department ensured that external goods meant to be delivered to the emergency department actually arrived as required. The auditor and the auditees discussed how far and by what means the department should evaluate their external suppliers, especially since the ISO 9001 certificate was addressed only to the emergency department and since the organizational internal structure would probably change. The auditor then finally stated for the auditees, “Now listen. I’m not supposed to be didactic – But, you will always be changing and the [quality management] system will need to change too.”

### Harmonizing the audit approach as imagined and done in standard and guidances, perceptions, and practices

Overall, a comparison between the standard and guidances for ISO 9001 certification processes, the certification body’s perceptions of the process and their practices showed an alignment towards characteristics of a prospective auditing approach in the auditing encounter. This meant an auditing approach enabling the recognition of opportunities for improvement, the provision of generic solutions, and the sharing of best practices.

## Discussion

In the following section we discuss the identified approaches to certification processes, and how these processes may support and nurture resilient performance in healthcare organizations undergoing certification.

### Certification as support for resilient performance

External assessment programs in healthcare can affect organizational and cultural changes, enhance support, collaboration and encourage improvement [4, 12–17], which may be fruitful in terms of resilience. However, the external assessment approaches that may support these changes are not well studied in healthcare. The standards and guidances for ISO 9001 certification audits explored in this study proposed an elasticity between consistent audits to identify possible nonconformities and audits that enhanced added value (e.g. recognizing support, education and improvement work). The certification regime left certification bodies, and hence auditors, with a latitude to navigate their auditing conduct towards the respective hospital context. The perceived and practiced auditing approaches were characterized by the auditors’ adaptation to the certification context. This included their interaction, negotiation, and dynamic communication with the auditees. These auditing characteristics seem to be in line with creation of reflexive spaces and responsiveness in the auditor – auditee encounter, that are important for creating conditions that nurture abilities for resilience in healthcare. Such reflexive spaces are characterized by trust, dialogue, respect and psychologically safe atmospheres [44].

The dynamic auditing approaches are possible since the ISO 9001 standard builds on generic requirements [28] that expands the auditor’s latitude to “translate” the requirements to specific organizational contexts. This means that auditors have a latitude that they can take advantage of. Auditors can adapt their performance to their perception of the maturity of the auditees’ quality management system and work. Such adaptation was emphasized by the certification auditing guidances. Generic requirements do also give the certified hospitals room to choose between different quality management tools that from their perspective meet the requirements the best. This means that when auditors try to communicate organizational challenges and requirements, such as managing risks, audit conclusions need to be constructed based on interactions, negotiation and dynamic communication practices with the auditees [45]. The certification body’s auditing approaches emphasized interactional audit processes. Further, the auditing practices inside hospital departments showed examples of the possibilities the auditors had to uncover challenges or opportunities for improvement of daily activity. Such practices can give surprises or external disruptions leading hospitals to trigger resilience by activating internal collective sensemaking processes and purposeful reorganizing [46–48].

As our theoretical approach we have presented four potentials proposed to be necessary for resilient performance; to *respond*, *monitor*, *learn* and *anticipate* [26]. These potentials are interdependent, and it can be challenging to operationalize them and keep them apart in healthcare research [49, 50]. E.g. when the auditors in this study performed audits in hospitals, they were monitoring the certified organization’s quality management system. But, such monitoring activity (the audit) does not necessarily mean that the hospital’s potential to monitor its own performance have been strengthened.

In our study we have identified characteristics of the organizations and their involvement in the certification supporting features of at least two of the four resilience potentials: the organization’s potential to respond and its potential to learn. Further explorations should be done related to all the potentials, here we will discuss more in depth the two most prominent in our study.

### The potential to respond

Healthcare organizations are often assumed to desire standardization and procedures [25]. To revisit and revise organizational action plans and procedures are important for diagnosing and improving the potential to respond, but not necessarily by including more detailed prescriptions or increasing the number of procedures [26]. There seems to be a general understanding of ISO certification as mostly concerned with formal prescriptions and procedures (often uncoupled from the organization’s own practices) [51, 52]. Our study revealed quite the opposite. The main concern of the certification body has been the complexity and high number of procedures in healthcare. Their effort has been on helping to reduce the extent of procedures, having focus on the functionality, appropriateness and availability of procedure for those working in the sharp end. Too many detailed checklists or procedures might undermine the discretion and autonomy of the health professionals working in hospitals [53, 54]. Procedures that support flexibility and adjustment, rather than constraining action and forcing people to make trade-offs, are important for resilient performance [54–57]. Similar results have been seen in previous research in hospitals [53], where a balance between structure and flexibility in standardization and employees’ discretion may improve performance and reduce errors.

It is noteworthy to see that the number of mandatory documented procedures required for a quality manual set out in the 2008 version of the ISO 9001 standard (included in this study) was reduced from six to none in the 2015 version. Now it emphasizes flexibility for the use of documented information required for the quality management system [58], and it stresses that ISO 9001 requires a “documented quality management system,” not a “system of documents” [59]. We recommend further studies of how this revised version can be conceptualized in a resilience perspective both from the certification body’s and the certified organization’s points of view. Also, studies from the patient’s and next of kin’s perspective could be of key value [60–62].

### The potential to learn

Two characteristics of ISO 9001 certification in our study are important for an organization’s potential to learn: the auditors’ opportunities and practices to affect learning during certification processes within organizations; and learning within or between organizational fields. Our assessment of the certification process describes a holistic approach to auditing in which the auditors used their scope of opportunities within the certification regime to share knowledge and make guidance for improvement part of their audits. We also found that auditors empowered local improvement initiatives at the sharp end. A holistic approach to auditing has been shown to be important for internal motivation and commitment to auditing practice and improvement work [18, 48]. The potential to learn is also exposed when auditors take initiatives to transfer experiences and good practices from one auditee to another, within or between organizational fields. That can be between different healthcare organizations and/or between the healthcare organizations and organizations in other sectors that undergo ISO 9001. Bringing these aspects into the certification process may create reflexive spaces where learning processes can take place between the certification body and the certified organization. In other studies, these reflexive spaces have been identified as keys in leveraging resilience into healthcare regulation and management [44].

The centralization of the knowledge that certification bodies provide may be important for resilient performance across an entire healthcare system [54]. Such centralized knowledge might be rules-of-thumb, but they could also be important lessons (challenges or opportunities) transmitted from one healthcare organization to another. In countries with widespread certification, certification bodies might be important mechanisms for spreading local information through their certification processes. The centralized knowledge bases that certification bodies serve and their influence on resilient performance remains to be studied in greater depth.

### Limitations

This study explores approaches to certification and discusses how these approaches might affect organizations. We have not studied how all the clauses in the ISO 9001 standard were assessed and translated during the auditing processes. Nor have we included the outcomes of different certification processes. Caution must therefore be taken about the relationship between the certification approach and process and the actual outcomes for the organizations.

According to the logic of representation and generalizability, the number of informants is small. Norway has only four certification bodies, and only a few lead auditors who perform ISO 9001 certifications in healthcare. The characteristics of the strategic participants are highly specific, and therefore strengthen the information power in the study [63]. To make theoretical generalizations from an in-depth case study, a theoretical sampling [36, 37] of a single institution (a certification body) was chosen instead of participants from several institutions.

Our study does not include the views from healthcare professionals and patients. This could add interesting insights to the certification research in a resilience perspective and should be further investigated.

## Conclusions

There is no simple way to manage and control quality improvement in healthcare. This study has shown that the auditing approach embedded in ISO 9001 certification expects certification bodies to have structures and systems in place to ensure consistent and objective certification processes. At the same time the normative references and guidance for auditors give a great deal of latitude for auditors to tailor their audits to context-specific problems, in order to add value to the auditees during certification audits. The members of a certification body perceived and practiced a flexible auditing approach in which both assessment and guidance were interwoven during certification audits. We argue that the ISO 9001 auditing approach described in this study supports resilient performance in healthcare through nurturing, especially the potential to respond and learn. We encourage further research to explore how certification processes contribute to the resilience potentials of anticipation and monitoring.

To date, certification has been questioned in Norway [64]. However, seen from a Norwegian context, there are key aspects from our study to highlight if one would like to scale up and implement a quality management certification in healthcare practice. Since it is not mandatory for a healthcare organization to be quality management certified, the benefits from a flexible approach, where organizations are supported to strengthen the already ongoing quality improvement work, should be emphasized. Moreover, focus on certification as one way of learning, monitoring and transferring experiences should also be highlighted as added value. In addition, the possible ability for certification processes to create reflexive spaces, discussions, and support to keep systems updated, could also contribute to motivating and supporting an upscaling of certification. An upscaling of certification programs needs to follow a thorough review and communication of research evidence on quality improvement processes and outcome from other countries and levels.

## Supplementary information

**Additional file 1.** Interview guide: Certification body - Lead auditors.

**Additional file 2.** Interview guide: Certification body – Managers and administrative personnel.

## Data Availability

The data generated and analyzed during this study are not publicly available due to concerns about confidentiality regarding a small sample size and the sensitive nature of the interviews and the certification body. Instead, quotations are included in the text. We will be happy to discuss the findings or the analysis if any questions should arise.

## Abbreviations

AAPG: Accreditation Auditing Practice Group
APG: Auditing Practice Group
ISO: International Organization for Standardization
WAI: Work as Imagined
WAD: Work as Done

## Footnotes

1 with quality management system (QMS) experts, auditors and practitioners drawn from the ISO Technical Committee 176 Quality Management and Quality Assurance (ISO/TC 176) and the International Accreditation Forum (IAF)

2 with accreditation experts, auditors and practitioners, drawn from the ISO Policy Committee for Conformity Assessment (ISO/CASCO), the ISO Technical Committee 176 Quality Management and Quality Assurance (ISO/TC 176) and the International Accreditation Forum (IAF).
