# Supplementary material for: Certification as support for resilience? Behind the curtains of a certification body — a qualitative study
Source: BMC Health Serv Res. 2020 Aug 8;20:730. doi: 10.1186/s12913-020-05608-5 (PMC7414657; doi:10.1186/s12913-020-05608-5)
Supplement: Supplementary file 1 — Additional file 1. Interview guide: Certification body - Lead auditors. [file 12913_2020_5608_MOESM1_ESM.pdf]

Interview guide: Certification body – Lead auditors

Part 1: About the informant and the organization and their approach to certification in hospitals according to ISO 9001

The informant
Position
Background and previous experience
Experience from audit work

What is your role and responsibilities related to hospital certification?
What do you believe the certification body consider as your most important tasks when it comes to certification?

Can you very briefly explain what certification according to ISO 9001 is?

Think back to your last audit in a hospital (health institution).
What do you see as your most important tasks during the audit?
Control, guidance, learning…
What do you perceive as your most crucial background and basis that enables you to carry out audits in hospitals?

- Professional background
- Another background
- Personal characteristics
- The standard
- Auditing system

Can you tell about how you are followed up by the certification body to be able to carry out your tasks as a lead auditor?

Imagine an axis where each extreme represents a different approach to auditing practice. One outer edge represents control, and the other represents guidance/advice. In what direction do you mainly feel that your auditing practice is going?

Can you tell more about what you think and understand about hospital certification?

- Third party control
- Regulatory Regime
- Control, audit, evaluation
- Guidance
- Learning, development
- Actors, Roles, functions, persons
- Management and management tools

(If you worked for the organization then) Can you think back to when you started talking about certification of quality systems in hospitals, and tell about that?
Who first introduced the idea?
Why was hospital certification considered?
Who was involved and drivers in the process?
What did you think about the idea of certification/accreditation in the health sector?

What do you think is the reason why the certification body conducts hospital certification?

Part 2: About the certification process and/or assessment and development of certification regimes

Think back on one of your latest certification audits in a hospital. Can you tell about how you planned and conducted the audit and what you emphasized during the process?
How were you involved?
How was the team put together?
How did you proceed / what methods did you use?
What methods do you use to communicate the requirements of the standard?
When you talked about certification in the opening meeting, what did you emphasize?

Interview guide: Certification body – Lead auditors

Whom do you perceive to be key players in the certification process?
What was the most crucial skill you needed in the process?
Were there any surprises / turning points?
Did you meet any resistance during the process?
Did you face other problems or challenges?
How did you handle resistance or challenges?
What could you possibly have done differently to avoid this?
If you were to do the whole process again, what would you have emphasized?

Can you tell about what you think the hospital has achieved through the certification process?
What are the most important achievements for the hospital from the standardization and certification process?
Are there any clear advantages and disadvantages for the certified hospital?
economic, legitimacy, visibility of quality systems…
How do you think others perceive the hospital after the certification?

Can you tell about something that has surprised you after you started with hospital certification?

If you were to give some advice to someone who wants to start with certification processes, what would you have emphasized then?

Part 3: On certification and regulation of quality and safety in hospitals

When you perform certification audits in hospitals and are asked the question "what is the purpose of certification?", What do you answer then?
What do you think are the most important arguments for hospital certification?
What do you think are the most important arguments against hospital certification?

A common argument for ISO 9001 certification is that the certificate itself is not an important matter, but that a certification process is a tool for continuous quality improvement and for operationalizing the requirements in the internal control regulation. Can you comment on this argument?

How do you perceive that ISO 9001 certification relates to the current regulation on quality and safety in hospitals?

- Internal control regulation
- Requirements in law and regulations
- The focus on professional soundness
- The focus on quality and safety work

It has been considered whether some form of certification or accreditation of hospitals should be required by law in Norway. How do you think that your ISO 9001 certification activities may be suitable in this context?
How do you understand certification as a way to regulate quality and safety in hospitals in the future?

How do you understand the Certification body's role and responsibilities if adverse events occur at a hospital/ward that you have certified?
How do you think you would understand this responsibility if the certifications you conducted were required by law?

If you should highlight some essential improvement points for hospitals certification practices, what would you emphasize?

If you should propose any changes to the ISO 9001 standard related to the health service, what would you emphasize?

We have now talked about quality and safety related to certification and the certification process, is there anything more you want to add that you have not told?
