# Supplementary material for: Certification as support for resilience? Behind the curtains of a certification body — a qualitative study
Source: BMC Health Serv Res. 2020 Aug 8;20:730. doi: 10.1186/s12913-020-05608-5 (PMC7414657; doi:10.1186/s12913-020-05608-5)
Supplement: Supplementary file 2 — Additional file 2. Interview guide: Certification body – Managers and administrative personnel. [file 12913_2020_5608_MOESM2_ESM.pdf]

Interview guide: Certification body – Managers and administrative personnel

Part 1: About the informant and the organization and their approach to certification in hospitals according to ISO 9001

The informant
Position
Background and previous experience
Experience from audit work

What is your role and responsibilities related to system certification?

Can you very briefly explain what certification according ISO 9001 is?
Does xxx have a specific definition of certification, and do you know this?
What do you think is the most important thing about the ISO 9001 standard?

Imagine an ordinary revision according to ISO 9001…
Can you tell about the most important tasks for a lead auditor in relation to certification?
Control, guidance, learning…
What do you perceive as your most crucial background and basis that enables you to carry out audits in hospitals?

- Professional background
- Another background
- Personal characteristics
- The standard
- Auditing system

Can you tell about what xxx does for lead auditors and technicians to be able to carry out the best possible certifications?

Imagine an axis where each extreme represents a different approach to auditing practice. One outer edge represents control, and the other represents guidance/advice. In what direction do you mainly feel that your auditing practice is going?

Can you tell more about what you think and understand about hospital certification?

- Third party control
- Regulatory regime
- Control, audit, evaluation
- Guidance
- Learning, development
- Actors, Roles, functions, persons
- Management and management tool

(If you worked for the organization then) Can you think back to when you started talking about certification of quality systems in hospitals, and tell about that?
Who first introduced the idea?
Why was hospital certification considered?
Who was involved and drivers in the process?
What did you think about the idea of certification/accreditation in the health sector?
What did you perceive to be the health sector's expectations for certification?

What do you think is the reason why the certification body conducts hospital certification?

Part 2: About the certification process and / or assessment of certification regimes

Think of an ordinary certification/audit process according to ISO 9001, preferably one you have been involved in. Can you tell about the steps in the process?
Are you usually involved in the process?
How is the team put together?
What methods do you use / how do you proceed?
What methods do you use to communicate the requirements of the standard?

Interview guide: Certification body – Managers and administrative personnel

Whom do you perceive to be key players in the certification work?
What is the most important competence you need in the process?
Do you know of any typical surprises/turning points?
If you encounter resistance, what are its characteristics?
Do you often face other problems and challenges?

Can you tell us a bit about what makes your ISO 9001 certification processes reliable?
What do you think the hospitals see as important for certifications to be reliable?

Can you tell about what you think hospitals/companies achieve through certification?
What do you think are the most important achievements?
Are there any clear advantages and disadvantages for hospitals/companies being certified?
economic, legitimacy, visibility of quality systems…
How do you think others perceive hospitals/companies after the certification?

Can you tell about something that has surprised you after you started with hospital certification?
What do you think is special about certification in hospitals in relation to other sectors?

If you were to give some advice to someone who wants to start with certification processes, what would you have emphasized then?

Part 3: On certification and regulation of quality and safety in hospitals

When you are on an assignment for xxx and are asked the question "what is the purpose of certification?", What do you answer then?
What do you think are the most important arguments for hospital certification?
What do you think are the most important arguments against hospital certification?

A common argument for ISO 9001 certification is that the certificate itself is not an important matter, but that a certification process is a tool for continuous quality improvement and for operationalizing the requirements in the internal control regulation. Can you comment on this argument?

How do you perceive that certification (according to ISO 9001) relates to the current regulation and control of quality and safety in hospitals?

- Internal control regulation
- Requirements in law and regulations
- The focus on risk management
- The focus on professional soundness
- The focus on quality and safety work

It has been considered whether some form of certification or accreditation of hospitals should be required by law in Norway. How do you think that your ISO 9001 certification activities may be suitable in this context?
How do you understand certification as a way to regulate quality and safety in hospitals in the future?

How do you understand the Certification body's role and responsibilities if adverse events occur at a hospital/ward that you have certified?
How do you think you would understand this responsibility if the certifications you conducted were required by law?

It is often pointed out that there is a lack of research that shows that certification affects quality in the health sector. Can you comment on this argument?

If you should highlight some essential improvement points for hospitals certification practices, what would you emphasize?

We have now talked about quality and safety related to certification and the certification process, is there anything more you want to add that you have not told?
